# Promoting civil discourse on social media using nudges: A tournament of seven interventions

**DOI:** 10.1093/pnasnexus/pgae380

**Published:** 2024-10-01

**Authors:** Tatiana Celadin, Folco Panizza, Valerio Capraro

**Affiliations:** Department of Economics, University Ca’ Foscari of Venice, San Giobbe, Venice 30121, Italy; Molecular Mind Laboratory, IMT School for Advanced Studies Lucca, Lucca 55100, Italy; Department of Psychology, University of Milan Bicocca, Milan 20126, Italy

**Keywords:** social media, harmful content, civil discourse, nudging, social norms

## Abstract

In this article, we test and compare several message-based nudges designed to promote civil discourse and reduce the circulation of harmful content such as hate speech. We conducted a large pre-registered experiment (*N* = 4,081) to measure the effectiveness of seven nudges: making descriptive norms, injunctive norms, or personal norms salient, cooling down negative emotions, stimulating deliberation or empathy, and highlighting reputation. We used an online platform that reproduces a social media newsfeed and presented the nudge as a message when entering the platform. Our findings indicate that none of the nudges significantly impacts participants’ engagement with harmful content. At the same time, nudges making descriptive norms salient selectively increase participants’ overall engagement with relatively harmless content. Additionally, making injunctive norms salient increased the likelihood of liking harmless posts. Exploratory text analysis also reveals that highlighting reputation leads to more substantial and coherent comments on harmful posts. These results suggest that nudges that activate norm considerations represent a promising approach to promoting civil discourse and making social media a safer and more inclusive space for all.

## Introduction

The spread of harmful content on social media, such as hate speech, has emerged as a pressing issue in contemporary society (1, 2). Typically directed at individuals based on characteristics such as ethnicity, sexual orientation, gender, social class, and physical appearance (3), hate speech and other forms of violent online expression can have severe consequences on the well-being of individuals, even if perpetrated by a minority of users (4). They can contribute to mental health issues, generating anxiety and fear (5, 6), and leading to social isolation (7), and can fuel discrimination and prejudice (8), thereby contributing to wider social divisions and conflicts (9). As also highlighted by the United Nations, it is of critical importance to identify interventions that can promote civil discourse and create safe spaces where users can express their ideas without fear of discrimination or harm (10).

Two widely used approaches to reduce the spread of harmful content and promote civil discourse are content moderation and counter-speech (7, 11). Moderation involves banning, suspending, or hiding comments and profiles that violate the terms and conditions of online platforms. Evidence suggests that content moderation can lead to a reduction of harmful content on those platforms (12–14). However, despite its effectiveness, moderation faces some limitations: banned users may migrate to less regulated platforms, and some harmful content may go undetected by the platforms’ algorithms (15, 16). On the other hand, counter-speech involves responding to harmful content with positive messages that aim to reduce negative behaviors (17). There is evidence that counter-speech does make users less prone to post harmful content (18–20). Yet, this approach is not easily scalable, and the most vulnerable targets of hate may be in a weak position to respond effectively (21).

A class of interventions with the potential to overcome some limitations of moderation and counter-speech involves nudging. Nudging offers a less invasive alternative to content moderation, as it does not alter users’ material payoffs. Moreover, it is more easily scalable than counter-speech because it relies on architectural changes within the platform (11, 22). Typically, nudging includes adjusting website architecture to promote prosocial behaviors online, such as compliance with the community norms and discouragement of bullying (23–25). A particularly relevant form of nudging, closely aligned with our research, involves the use of targeted messages. These message-based nudges are cost-effective, requiring minimal implementation resources while potentially exerting a significant impact. For instance, displaying normative information about community rules during user interactions increased rule compliance in a large-scale field experiment (26). Nudges that elicited empathy and notified users about the potential implications of their posts were effective at promoting empathic responses to instances of cyberbullying (27). Informing users about the audience for their actions enhanced their sense of responsibility and increased the likelihood of flagging cyberbullying posts (28).

A crucial step towards the implementation of nudging interventions is the assessment of their relative impact; while specific nudges may be effective, conducting cost–benefit analyses is essential to understanding the most promising interventions (29). Yet, to our knowledge, there is a shortage of studies comparing different nudges aimed at reducing the spread of harmful content and promoting more civil conversations in the same social media environment. Our study aims to reduce this gap by examining the efficacy of seven distinct message-based nudges. Following the emerging literature using intervention tournaments (30, 31) or megastudies (29, 32, 33), we test multiple interventions on the same participants pool, allowing a uniform comparison of their relative impacts.

To this end, we selected seven message-based nudges aimed at reducing the spread of harmful content and promoting civil behavior: making descriptive norms, injunctive norms, or personal norms salient, cooling down negative emotions, stimulating deliberation or empathy, and highlighting reputation. We focused on these specific nudges because an extensive body of literature has shown that they can promote prosocial behavior in a broad variety of social contexts. For example, descriptive norms nudges can increase vaccination rates and promote advocacy for vaccination (34). Injunctive norms nudges can increase the reporting rates of fake news (35). Making personal norms salient can heighten generosity and cooperative behavior, with effects persisting in subsequent decisions (36). Encouraging deliberation can increase intentions to wear a face mask during a pandemic (37). Inducing empathy can favor social distancing and mask wearing (38) as well as positive attitudes toward political outgroups (39). Social motivations such as reputation increase the accuracy of the assessment of online information (40, 41). Alerts about potentially emotionally charged content can decrease the likelihood of sharing offensive material (22). In sum, these nudges have been proven to encourage prosocial behavior in various contexts, including in, but not limited to, social media interactions. Therefore, given that reducing engagement with harmful posts and increasing engagement with nonharmful posts can be considered forms of prosocial behavior, we hypothesized that each of these interventions could interact with the harmfulness of the content in determining engagement levels. We do not have a priori hypotheses on their relative effectiveness; the objective of this study is to determine which intervention is most effective.

**Fig. 1.**
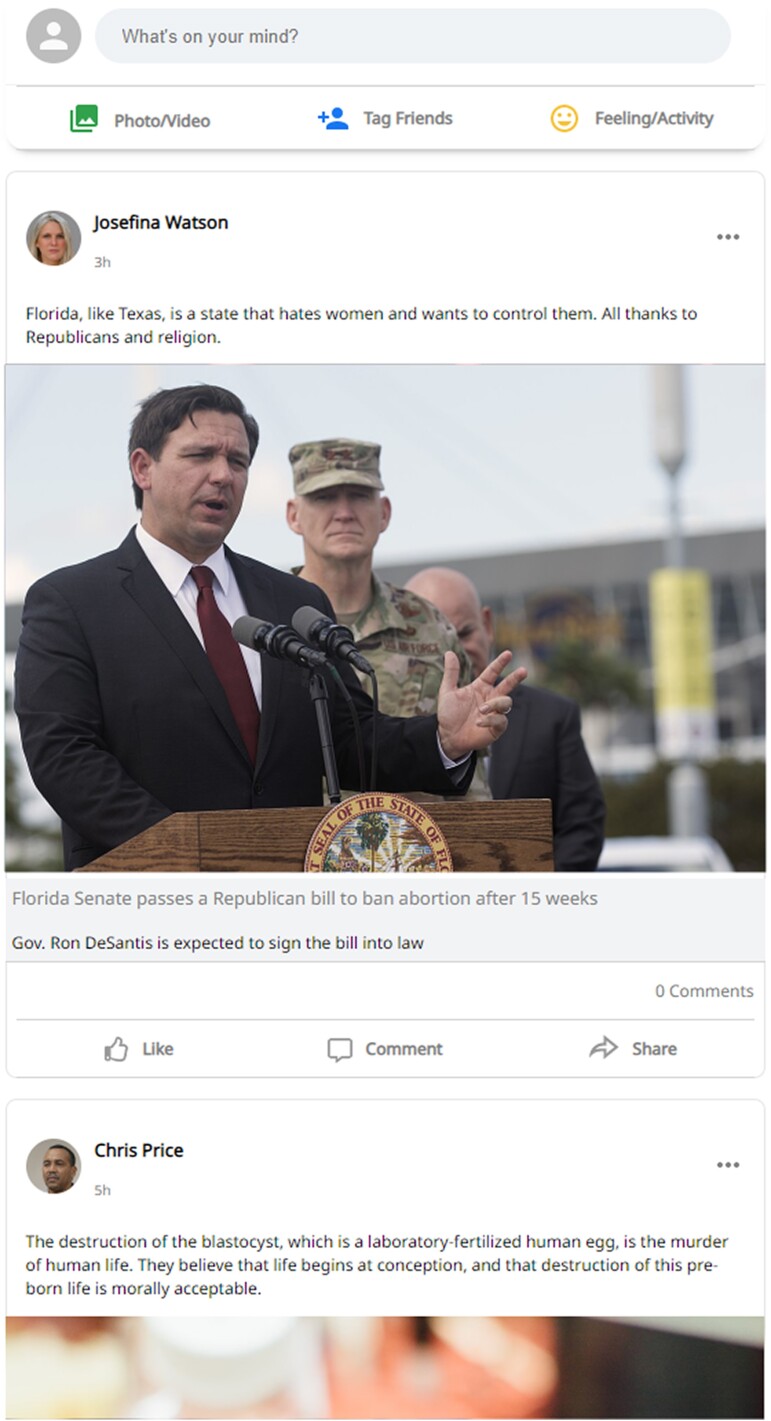
Sample of the Facebook’s newsfeed used in the experiment. Names and profile pictures of all posts are not real but were randomly generated for research purposes through behindthename.com/random and generated.photos.

**Fig. 2.**
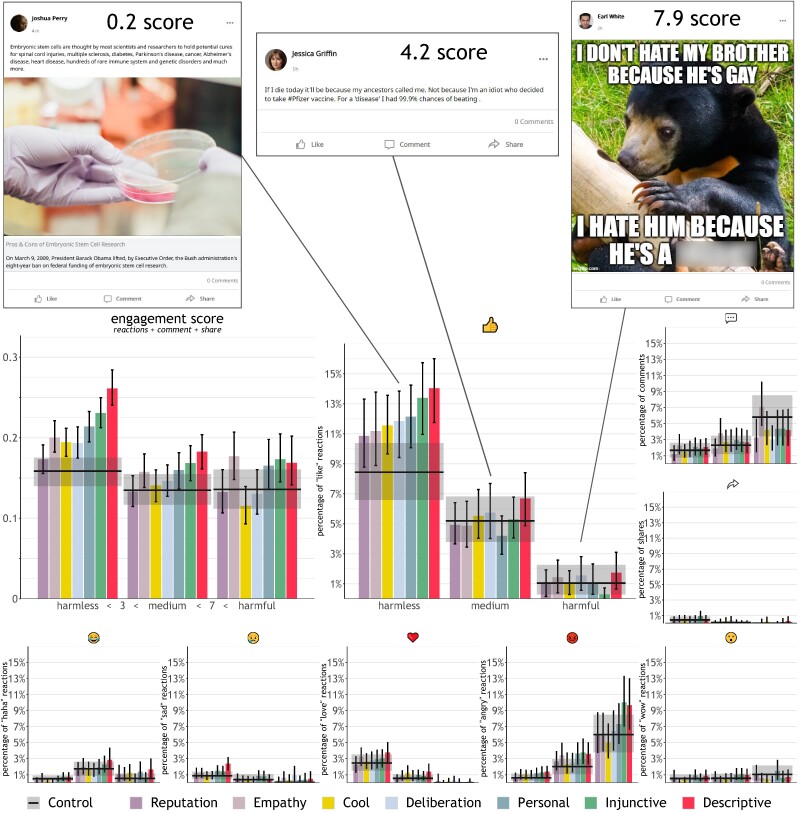
Top: three sample posts from the newsfeed, each annotated with its harmfulness score. The content of the highly harmful post is obscured but was fully readable during the experiment. Disclosure: This figure contains offensive language, which neither NAS nor the authors condone. Bottom, clockwise from the top left: composite engagement score, frequency of “like” reactions, and frequency of the other reactions. These metrics are averaged across both participants and posts with comparable ranges of harmfulness. Posts are divided into three levels for illustrative purposes. In the regressions, harmfulness is treated as a continuous variable. The colored bars represent the average metrics for each experimental condition, whereas the black horizontal lines denote the control group averages. Error bars and the shaded regions around the averages represent confidence intervals adjusted for multiple comparisons using Bonferroni-corrected bootstrap methods.

We recruited 4,081 participants living in the United States through the online recruiting platform Prolific and randomly assigned them to one of eight conditions, including seven message-based nudge interventions and a control group with no-intervention (between-subjects design). Participants in all conditions except the control group were presented with a message, similar to those used in previous studies (42). In the control group, no nudge was shown. The exact wording of each nudge, along with the number of participants per condition and a brief definition of each nudge, can be found in Table 1. Then, all participants were redirected to a platform, called Mock Social Media Website Tool, that faithfully reproduces Facebook’s newsfeed (43). This choice aims at increasing the study’s ecological validity, in line with previous work (27, 28, 44, 45). Participants in each condition interacted with the newsfeed in a manner akin to real social media usage, scrolling through various posts with the option to engage by sharing, commenting, or reacting. The sum of all the actions taken by the participant with each post represents our measure of engagement, which serves as our primary dependent variable. Detailed visualization of the newsfeed can be found in Fig. 1. In the newsfeed, participants were shown 14 posts of varying degrees of harmfulness randomly drawn from a larger pool of 49 posts. The level of harmfulness of each post was determined through an out-of-sample survey where 201 participants rated each post on a scale from 0 to 10 (see Fig. 2 for examples, and osf.io/tsxk2 for the full collection of posts). The mean harmfulness of the posts was 3.68 (SD = 2.72), with a minimum of 0.17 and a maximum of 9.67. The topics for these posts spanned a range of contentious issues, including: abortion, assisted suicide, gun control, marijuana legalization, politics, science (animal testing, climate change, stem cells, vaccination), and social justice (gender equality, LGBTQIA+, racism). Further details about the experimental procedure can be found in the Methods section. Descriptive statistics, including means, standard deviations, minima, and maxima for each condition and reaction, are reported in Table S1.

**Table 1.** Exact wording of the seven message-based nudges.

Nudge	Definition	Wording
Descriptive norm (N=481)	Making salient what others regularly do.	Sometimes people make decisions taking into account what they believe other people would do in the same context. Other times, people make decisions by ignoring what they believe others would do. Many people believe that considering others’ expected actions leads to good decision-making. **When we take into account what others would do, we make decisions that are typically socially accepted and widespread.** Please **make your decisions** on this social media platform by **taking into account what you believe others would do**.
Injunctive norm (N=533)	Making salient what others approve or disapprove of.	Sometimes people make decisions taking into account what other people would approve or disapprove of. Other times, people make decisions by ignoring what others consider to be the right thing to do. Many people believe that considering what others approve or disapprove of leads to good decision-making. **When we take into account what others approve or disapprove of, we make decisions that are typically well-regarded.** Please **make your decisions** on this social media platform by **taking into account what you believe others would approve or disapprove of**.
Personal norm (N=516)	Making salient what one personally believes to be the right thing to do.	Sometimes people make decisions taking into account what they think is the morally right thing to do. Other times, people make decisions by ignoring their internal sense of right and wrong. Many people believe that considering their internal morality leads to good decision-making. **When we take into account what we believe to be the right thing, we make decisions that are typically in line with our deepest beliefs**. Please **make your decisions** on this social media platform by **relying on what you think is the morally right thing to do**.
Negative emotions (N=527)	Regulating one’s emotions to cool down immediate negative feelings.	Sometimes people make decisions following their immediate negative emotions. Other times, people make decisions by letting their emotions cool down first. Many people believe that avoiding their immediate negative emotions leads to good decision-making. **When we avoid our immediate negative emotions, we make decisions that typically prevent us from feeling bad.** Please **make your decisions** on this social media platform by **letting your negative emotions cool down**.
Deliberation (N=511)	Engaging in a slower and more deliberative mode of thinking.	Sometimes people make decisions taking into account what they think is the rational thing to do. Other times, people make decisions by ignoring their logic and reason. Many people believe that considering their rational side leads to good decision-making. **When we take into account our analytic part, we make decisions that are typically well-thought.** Please **make your decisions** on this social media platform by **taking into account what you think is the rational thing to do**.
Empathy (N=501)	Placing oneself in another person’s situation.	Sometimes people make decisions taking into account the point of view of the other people involved. Other times, people make decisions by ignoring the point of view of others. Many people believe that putting oneself in the shoes of others leads to good decision-making. **When we take into account what other people experience from their perspective, we make decisions that are typically empathic.** Please **make your decisions** on this social media platform by **taking into account the point of view of others**.
Reputation (N=494)	Focusing on the opinion that others have about oneself.	Sometimes people make decisions taking into account how these decisions will affect their own reputation. Other times, people make decisions by ignoring their effect on reputation. Many people believe that considering how their decisions will impact their own reputation leads to good decision-making. **When we take into account that our actions are judged by others, we make decisions that are typically well-evaluated.** Please **make your decisions** on this social media platform by **taking into account your reputation**.
Control (N=518)		No nudge was shown.

Messages were displayed on the screen just before participants entered the social media newsfeed.

## Results

We begin by examining the overall engagement, defined as the sum of all possible actions (reactions, comments, shares) taken by the participants. As pre-registered, we conduct a linear regression with robust standard errors clustered at the participant and post levels. As regressors, we include the harmfulness of the post, seven dummies, one for each intervention, and the seven interactions between each of the intervention dummies and the harmfulness of the post:


$$\begin{array}{rl} y_{ij} & =\alpha +\beta_{1}\cdot {Harmfulness}_{\;j}+\sum_{k=1}^{7}\gamma_{k}\cdot {Condition}_{ik}\\ & \;+\sum_{k=1}^{7}\delta_{k}\cdot ({Harmfulness}_{\;j}\times {Condition}_{ik})+\epsilon_{ij} \end{array}$$

where $y_{ij}$ is the total engagement of subject *i* for post *j*; ${Harmfulness}_{\;j}$ is the harmfulness value of post *j*; ${Condition}_{ik}$ is a dummy variable that takes value 1 if subject *i* participates in condition *k*. Our key variables of interest are the seven interactions, which measure how the difference between overall engagement in the corresponding intervention and overall engagement in the control group varies when the harmfulness of the post increases. We find that this interaction is significant and negative when the nudge is based on descriptive norms (β=−0.011, t=−3.66, P=0.001), injunctive norms (β=−0.007, t=−2.63, P=0.012), deliberation (β=−0.006, t=−2.07, P=0.044), and cooling down negative emotions (β=−0.009, t=−3.23, P=0.002). The effects of descriptive norms and cooling down negative emotions are robust to Bonferroni correction.

These results show that as the harmfulness of posts increases, the differences between overall engagement in the interventions and overall engagement in the control group decrease. This trend could be explained by one of two non-mutually exclusive mechanisms: (i) the interventions decrease engagement with relatively harmful posts or (ii) the interventions increase engagement with relatively harmless posts. To determine which of these two mechanisms is at work, as an exploratory analysis, we look at the simple effects of the interventions. This analysis allows us to estimate the interventions’ effects separately for posts with extreme values of harmfulness.

For extremely harmless posts, the model estimates significantly higher engagement in all interventions, compared to the control group, with the exception of the reputation nudge. This increase is robust to Bonferroni correction for personal (β=0.059, t=3.09, P=0.003), descriptive (β=0.114, t=4.99, P<0.001), and injunctive (β=0.078, t=3.88, P<0.001) norm nudges. According to the model’s predictions, for every 10 posts, participants in the control group engage an average of 1.65 times (95% CI=[1.28,2.01]). The engagement rate increases to 2.24 times (95% CI=[1.84,2.63]) with a personal norm message, 2.42 times (95% CI=[2.02,2.83]) with an injunctive norm message, and 2.77 times (95% CI=[2.26,3.29]) with a descriptive norm message.

For extremely harmful posts, engagement levels appear to be closely aligned across conditions. For instance, the model estimates 1.15 engaged posts per 10 in the control group scenario (95% CI=[0.72,1.58]), closely matched by 1.14 engaged posts in the descriptive norm condition (95% CI=[0.66,1.62]).

Contrary to our pre-registered primary hypothesis, these analyses suggest that the channel through which the descriptive norm intervention works is not by decreasing engagement with more harmful posts, but rather by increasing engagement with relatively harmless posts. On the other hand, the cooling down negative emotions intervention likely works through a combination of two non-significant effects: an increase in engagement with harmless posts and a decrease in engagement with harmful posts. These conclusions are exemplified in Fig. 2, “engagement score” panel, where, for simplicity, we categorized the posts into three groups, according to their level of harmfulness.

Next, as pre-registered, we examine the individual reactions to understand which reactions drive these changes in engagement, starting with the most common reaction: liking a post. To this end, we conduct a logit regression with robust standard errors clustered at the participant and post levels. As before, we include the harmfulness of the post, seven dummy variables for each intervention, and seven interactions between each intervention dummy and the harmfulness of the post as regressors. Our key variables of interest are the seven interactions. We find significant negative interactions for nudges based on personal norms (β=−0.122, z=−2.49, P=0.013), descriptive norms (β=−0.073, z=−2.07, P=0.039), and injunctive norms (β=−0.160, z=−4.02, P<0.001). The effect of nudging the injunctive norms is robust to Bonferroni correction.

Examining the simple effects, participants in the control group are predicted to like an average of 1.27 out of every ten harmless posts (95% CI=[1.12,1.43]), compared to 2.16 posts in the injunctive norm intervention (95% CI=[1.96,2.36]). For extremely harmful posts, the predicted liking rates are very low across all conditions and never significant after Bonferroni correction. This suggests that the injunctive norm intervention works primarily by increasing the liking of harmless posts rather than decreasing the liking of harmful ones. See Fig. 2, “like” panel.

We then investigate the other reactions – love, laugh, anger, cry, and wow – as well as commenting and sharing. Using logit regressions, we account for the harmfulness of the post, intervention groups, and their interactions. We observe a general decrease in “love” reactions, juxtaposed with a rise in reactions of “anger”, “wow”, and in commenting behavior (center-right and bottom center in Fig. 2). This shift from positive to more contentious or surprised reactions serves as a compensatory mechanism, preventing engagement levels from plummeting. On the other hand, the interactions between the intervention dummies and harmfulness are never significant after Bonferroni correction. In sum, nudging interventions affect engagement primarily through changes in the frequency of “likes”. We refer to the Section S2, for full regression tables.

As a robustness check, we investigate whether respondent-level factors such as gender, age, and political orientation might moderate the efficacy of the interventions. We found significant moderation only in the context of the post’s topic. Specifically, the effectiveness of the intervention aimed at cooling down negative emotions was predominantly evident in posts concerning assisted suicide and gun control. Similarly, the descriptive norm nudge showed a more pronounced influence on posts related to assisted suicide, gun control, legalization, and politics. See Sections S3–S6, for regression tables.

As the final phase of the analysis, we conducted a series of exploratory text analyses to test for differences in comment style across conditions. To analyze this textual data, we use PeRspective API, a tool developed by Google Jigsaw, which uses pretrained machine learning algorithms to analyze conversational content (46). Each comment (total N=1,507) was thus weighted against a variety of metrics, and normalized on a scale from 0 to 1 (see Section S7, for the entire list of metrics including a definition for each). We run a series of mixed-effects linear regressions, one for each metric, treating these metrics as dependent variables. The models include the seven intervention dummies, the level of harmfulness of the post, and their interaction, as predictor variables. Standard errors are clustered at the participant and post levels. Once again, our variables of interest are the interaction terms. We find no statistically significant differences for most of the metrics tested, with the exception of two metrics: “unsubstantial”, and “incoherent”. For these two metrics, we find significant interactions between experimental condition and harmfulness score in most interventions (see Tables S7–S9, for regression tables). In both cases, the interaction is robust to Bonferroni correction only in the reputation condition (substance: β=0.249, t=3.86, P<.001; coherence: β=0.298, t=3.70, P<.001). Note, however, that these two metrics are strongly correlated (Pearson correlation coefficient: r(1,563)=0.312, t=12.95, P<0.001). Essentially, as the harmfulness of a comment increases, participants subjected to the reputation condition are more likely to leave more substantial and coherent responses compared to participants in the control group. An illustrative example of a substantial and coherent comment left by one participant is: “I’m not necessarily an advocate of any mind altering substances, however when you break it down to risks, health concerns, and other aspects I feel like the laws to allow people to drink alcohol but not consume marijuana contradict themselves.”).

## Discussion

We tested seven message-based nudges designed to reduce the spread of harmful content and promote a more civil discourse on social media. Our findings indicate none of the nudges reduce the spread of harmful content. However, a nudge making descriptive norms salient increases participants’ overall engagement with harmless content and a nudge making injunctive norms salient increases the likelihood of participants liking harmless posts.

Social media interventions may work through two distinct, although not mutually exclusive, mechanisms: reducing interactions with harmful content or boosting interactions with harmless content. Since the vast majority of online content is harmless, some scholars have argued that increasing engagement with harmless content is as important, if not more so, than reducing engagement with harmful content (47). This is because the ratio of harmless to harmful content, which is the essential factor defining the overall quality of online content, would be more strongly impacted (13, 48). Moreover, increased engagement with harmless content could have the effect of amplifying this content even more, as the most popular content is prioritized by ranking algorithms. From this perspective, one of the positive aspects of these interventions is that they work precisely through this mechanism.

Understanding why these interventions appear to work primarily through this mechanism is an interesting direction for future work. At this stage of research, we can only speculate. Looking at Fig. 2, one may notice some promising trends for harmful content. The personal, descriptive, and injunctive norm nudges seem to increase the angry reaction to harmful posts. Moreover, most interventions appear to reduce the frequency of comments. However, these trends do not reach common thresholds of statistical significance. This may be due to the limited power of this study to detect significant effects for less used reactions. In other words, it is possible that the null effects of the interventions on harmful posts stem from the combination of two “socially positive” effects: one that leads people to react more angrily to harmful posts, and another that encourages people to ignore harmful posts and avoid commenting on them. Future experiments with a much larger sample size can illuminate this point. Regardless of the outcomes of these experiments, it is important to note that the overall positive effect on engagement of the descriptive norm and cooling down negative emotions interventions is promising from a practical perspective. It has been argued that social media platforms have a tendency to maximize engagement, even at the cost of promoting harmful content (49). From this perspective, it is encouraging that these interventions increase engagement while promoting harmless content.

We also found that the nudge aimed at cooling down negative emotions interacts with the harmfulness of the posts in the predicted direction. The analysis of simple effects provides evidence that this intervention likely operates through a combination of two mechanisms: increasing engagement with harmless posts and decreasing engagement with harmful posts. However, none of these effects was singularly significant, possibly due to the limited power of our study. Future work could investigate more thoroughly the capacity of this specific nudge intervention to symmetrically affect engagement for both harmful and harmless posts.

A strength of message-based interventions lies in their scalability, which stands in contrast to the resource-intensive nature of counter-speech strategies and content moderation by human reviewers. Message-based nudges can be easily integrated via architectural changes within a platform. Furthermore, their implementation can be recurrent, using reminders like pop-ups when users return on a social media platform after a period of inactivity. Nonetheless, message-based nudges are not without their limitations. One concern regards their modest impact, especially when compared to more significant structural modifications to a platform. For instance, one study demonstrated that introducing a button to flag misinformation reduced the sharing of such content by 25%, whereas an accuracy nudge resulted in only a 5% decrease (50). In this regard, it is important to note that the effect sizes for the most successful interventions in our study were substantial. The total engagement rate in the descriptive norm condition was 68% higher than in the control group. Similarly, the average number of likes in the injunctive norm condition rose by 70%, compared to the control group.

Another set of concerns involves the possibility that the effectiveness of message-based nudges may decrease over time (51). Moreover, especially if the nudges are repeated too frequently, there is the potential for user desensitization. Future work should explore the boundary conditions of these specific nudges, bearing in mind that addressing a complex challenge like the reduction of harmful content likely requires more than a single type of intervention. Message-based nudges should not be regarded as the definitive solution, but as one tool among many in a comprehensive strategy aimed at promoting a safer and more respectful environment. From this perspective, another promising avenue for future research is exploring how message-based nudges can be employed synergistically with other interventions to create a more cohesive and effective approach.

Moreover, since we tested our interventions on a platform that reproduces Facebook’s newsfeed, it would be interesting to see whether our results extend to other social media platforms. Additionally, our design did not fully replicate a social media environment where individuals can actively interact, which could have influenced the use of some reactions, such as commenting and sharing. Thus, another direction for future research would be to explore how these interventions would perform in a field experiment.

Our conclusive exploratory text-analysis revealed that participants exposed to a message about the consequences for their reputation tended to write more substantial and coherent comments, in response to harmful posts. This trend was observed also in several other interventions, but it was not robust to Bonferroni correction. It is important to acknowledge, however, that these findings, derived from a subset of participants, may not be sufficiently powered to draw definitive conclusions. Moreover, the metrics for evaluating the substance and coherence of comments were originally trained on the content of a single newspaper (The New York Times) and may be biased by the readership of that journal. Additionally, due to technical constraints, some lengthy messages (N=36) exceeding 255 characters were truncated in our dataset. In this case, the analyses were based solely on the available portions of these messages. Future research should employ preregistered, more powerful designs to investigate in greater detail how message-based nudges influence commenting style.

Overall, these results suggest that some message-based nudges, and in particular those activating normative considerations, could help create a more positive and inclusive online environment. Future work should investigate the mechanisms through which these interventions work and their boundary conditions.

## Methods

### Procedure

This study was approved by the Middlesex University Ethics Committee n. 21556. Pre-registration and data are available at: osf.io/tsxk2

We selected 71 posts from different platforms (e.g. Facebook, Twitter, Reddit, 4chan). We recruited 201 participants through Prolific, who provided informed consent to participate in the study, to rate the harmfulness of these posts along two dimensions:

- “How abusive do you think this post is?”;
- “How hateful do you think this post is?”.

Since the two dimensions were consistently correlated (mean by-post Cronbach’s α=0.80), we aggregated them into a single *harmfulness* index. Some posts were excluded to make the levels of harmfulness as heterogeneous as possible. In addition, some posts were discarded because there were too many conservative-leaning posts with high values of harmfulness. The exclusion was done by random sampling so that the selection of posts could not be biased by the researchers. Due to an error in pairing posts with users, a post describing an abortion experience was mistakenly paired with a male avatar. The error was discovered after data collection had begun and resulted in the exclusion of 606 participants who viewed the post, leading to the second data collection. Thus, the final set of stimuli contained 49 posts: 27 were conservative, 22 were progressive; on a scale of 0 to 10, the mean harmfulness was 3.68 (SD = 2.72), with a minimum of 0.17 and a maximum of 9.67.

Once we collected the harmfulness ratings of the posts, we ran the main experiment. We recruited 4,081 participants from the United States through Prolific and randomly assigned them to one of eight conditions, including seven nudges and the control group (between-subjects design). Specifically, we ran two sessions. In the first session (2022 September 12), we collected N=1,442 subjects, and in the second session (2022 October 6–8), we collected N=2,639 subjects. We invited participants who declared, in the Prolific prescreening, that they regularly use social media. Participants were shown 14 posts of varying degrees of harmfulness. To increase ecological validity, we used a platform, called Mock Social Media Website Tool, that faithfully reproduces Facebook’s newsfeed (43). Names and profile pictures provided in the Facebook newsfeed were randomly generated online and for research purposes only through behindthename.com/random and generated.photos. The macro-topics of the posts were: abortion, assisted suicide, gun control, legalization, politics, science, and social justice. The science macro-topic included posts on animal testing, climate change, stem cell, and vaccination; the social justice macro-topic included posts on gender equality, LGBTQIA+, and racism. Participants in each condition could interact with the posts by sharing, commenting, or reacting to them. Before accessing the newsfeed, all the conditions except the control group presented participants with a nudge message. The messages can be found in Table 1.

### Sample size estimation and sensitivity analysis

The sample size was determined based on our budget constraints. We did not perform an a priori power analysis. However, we present here a sensitivity analysis for our main effect of interest: the interaction between treatment and harmfulness. As an example, we consider total engagement as the dependent variable and the descriptive norm nudge as the treatment. The other estimations are very similar, as there are only minor changes in the numerical specifications that follow. Our main analysis was conducted using 14 observations (posts) per participant over approximately 1,020 participants for each contrast, for a total of approximately 14,000 observations per analysis. We compute the minimum detectable effect size given the $R^{2}$ of the reduced model (without the interaction term, 0.0053), the number of observations (n=14,476), the number of control covariates (including fixed effects, n=1,083), an alpha of 5%, and different levels of power. Our results report that the model is able to detect an increase in the total variance explained by 0.05pp for beta = 80%, by 0.07pp for beta = 90%, and by 0.09pp for beta = 95%.

## Supplementary Material

pgae380_Supplementary_Data

## Data Availability

Pre-registration, data, analysis code, and materials have been deposited in https://osf.io/tsxk2/. ChatGPT was used to polish the text.

## Preprints

A preprint of this article is published at: http://dx.doi.org/10.2139/ssrn.4776172.
