# Supplementary material for: Promoting civil discourse on social media using nudges: A tournament of seven interventions
Source: PNAS Nexus. 2024 Oct 1;3(10):pgae380. doi: 10.1093/pnasnexus/pgae380 (PMC11443661; doi:10.1093/pnasnexus/pgae380)
Supplement: pgae380_Supplementary_Data [file pgae380_supplementary_data.pdf]

Supplementary Materials
Promoting civil discourse on social media using nudges: A tournament of seven interventions

Tatiana Celadin, Folco Panizza, and Valerio Capraro

Contents

1 Descriptives **2**

2 Main Analysis **3**

3 Overall Engagement: robustness check for topics **4**

4 Like: robustness check for topics **6**

5 Demographics: robustness check **8**

6 Political orientation: robustness check **10**

7 Text Analysis **12**

7.1 Definition of the metrics 12

7.2 Analysis 12

1 Descriptives

Condition	Angry	Comment	Haha	Like	Love	Sad	Share	Wow	Engagement
Control	0.29	0.37	0.14	0.85	0.20	0.07	0.03	0.09	1.94
	(0.88)	(1.34)	(0.57)	(1.61)	(0.65)	(0.35)	(0.25)	(0.49)	(3.30)
	[0,5]	[0,14]	[0,5]	[0,14]	[0,5]	[0,5]	[0,4]	[0,6]	[0,14]
Cool	0.29	0.32	0.13	1.07	0.25	0.09	0.05	0.06	2.13
	(0.92)	(1.14)	(0.50)	(1.87)	(0.75)	(0.40)	(0.35)	(0.34)	(3.47)
	[0,9]	[0,10]	[0,5]	[0,12]	[0,5]	[0,4]	[0,5]	[0,5]	[0,14]
Deliberation	0.36	0.33	0.11	1.08	0.23	0.10	0.05	0.06	2.18
	(1.10)	(1.21)	(0.43)	(1.79)	(0.83)	(0.36)	(0.32)	(0.37)	(3.44)
	[0,9]	[0,13]	[0,3]	[0,14]	[0,8]	[0,4]	[0,3]	[0,6]	[0,14]
Descriptive	0.50	0.36	0.21	1.28	0.32	0.20	0.05	0.12	2.86
	(1.25)	(1.20)	(0.62)	(1.73)	(0.83)	(0.55)	(0.26)	(0.52)	(3.95)
	[0,8]	[0,12]	[0,5]	[0,10]	[0,6]	[0,4]	[0,3]	[0,6]	[0,14]
Empathy	0.40	0.50	0.16	1.02	0.25	0.11	0.04	0.05	2.39
	(1.25)	(1.65)	(0.56)	(1.61)	(0.71)	(0.41)	(0.29)	(0.26)	(3.67)
	[0,10]	[0,14]	[0,5]	[0,13]	[0,5]	[0,4]	[0,3]	[0,3]	[0,14]
Injunctive	0.52	0.36	0.17	1.17	0.24	0.14	0.07	0.11	2.61
	(1.45)	(1.32)	(0.54)	(1.74)	(0.69)	(0.50)	(0.30)	(0.46)	(3.90)
	[0,10]	[0,11]	[0,4]	[0,8]	[0,6]	[0,4]	[0,3]	[0,4]	[0,14]
Personal	0.45	0.39	0.16	1.04	0.24	0.15	0.05	0.14	2.47
	(1.23)	(1.30)	(0.56)	(1.57)	(0.69)	(0.49)	(0.28)	(0.60)	(3.69)
	[0,9]	[0,12]	[0,4]	[0,9]	[0,6]	[0,4]	[0,3]	[0,6]	[0,14]
Reputation	0.34	0.26	0.13	0.98	0.17	0.11	0.04	0.11	2.01
	(1.09)	(1.22)	(0.48)	(1.67)	(0.61)	(0.39)	(0.22)	(0.52)	(3.39)
	[0,11]	[0,13]	[0,4]	[0,14]	[0,6]	[0,3]	[0,2]	[0,6]	[0,14]

Table S1: Descriptive statistics: For each condition and each reaction, we report the mean, the standard deviation (in round brackets), and the minimum and maximum values (in square brackets).

2 Main Analysis

	Model 1 Engagment	Model 2 Like	Model 3 Angry	Model 4 Laugh	Model 5 Love	Model 6 Cry	Model 7 Wow	Model 8 Sharing	Model 9 Comment	Model 9 Reactions
Harmful	-0.005	___-0.272***___	___0.324***___	0.070	___-0.521***___	-0.150*	0.132*	___-0.490***___	___0.206***___	___-0.100***___
	(0.003)	___(0.038)___	___(0.037)___	(0.052)	___(0.102)___	(0.075)	(0.066)	___(0.135)___	___(0.037)___	___(0.024)___
Personal	___0.059**___	___0.442**___	0.667*	-0.127	0.040	0.538*	0.788	0.159	0.289	___0.359**___
	___(0.019)___	___(0.144)___	(0.323)	(0.295)	(0.286)	(0.225)	(0.440)	(0.542)	(0.356)	___(0.122)___
Cool	0.047*	_0.368**_	0.450	-0.416	-0.023	-0.162	0.064	0.036	0.176	0.315*
	(0.019)	_(0.128)_	(0.341)	(0.249)	(0.278)	(0.422)	(0.481)	(0.484)	(0.355)	(0.128)
Reputation	0.017	0.274*	-0.119	-0.410	-0.244	0.292	0.293	0.245	-0.161	0.109
	(0.019)	(0.132)	(0.331)	(0.376)	(0.274)	(0.221)	(0.529)	(0.417)	(0.416)	(0.130)
Descriptive	___0.114***___	___0.596***___	0.689*	0.345	0.358	___1.116***___	0.932	-0.159	0.412	___0.654***___
	___(0.023)___	___(0.133)___	(0.322)	(0.294)	(0.270)	___(0.254)___	(0.540)	(0.520)	(0.364)	___(0.123)___
Injunctive	___0.078***___	___0.639***___	0.508	0.268	0.093	0.494	0.573	0.481	0.193	___0.462***___
	(0.020)	___(0.137)___	(0.281)	(0.225)	(0.257)	(0.348)	(0.455)	(0.432)	(0.331)	___(0.125)___
Empathy	0.041*	0.311*	0.467	-0.054	0.211	0.240	-0.774	-0.108	0.395	0.257
	(0.020)	(0.157)	(0.296)	(0.398)	(0.267)	(0.218)	(0.470)	(0.444)	(0.357)	(0.132)
Deliberation	0.040*	0.332*	0.298	-0.513	-0.035	-0.289	-0.220	-0.031	0.408	0.218
	(0.020)	(0.136)	(0.303)	(0.301)	(0.339)	(0.380)	(0.583)	(0.530)	(0.370)	(0.124)
Personal×Harmful	-0.005	-0.122*	-0.028	0.064	0.083	0.057	-0.077	0.135	-0.043	-0.014
	(0.003)	(0.049)	(0.039)	(0.042)	(0.107)	(0.069)	(0.077)	(0.176)	(0.048)	(0.024)
Cool×Harmful	___-0.009**___	-0.065	-0.076	0.072	0.146	0.114	-0.095	0.249	-0.061	-0.054*
	___(0.003)___	(0.037)	(0.052)	(0.042)	(0.092)	(0.100)	(0.088)	(0.173)	(0.044)	(0.026)
Reputation×Harmful	-0.003	-0.064	0.047	0.070	0.055	0.022	-0.021	-0.079	-0.037	0.003
	(0.003)	(0.038)	(0.040)	(0.057)	(0.103)	(0.062)	(0.076)	(0.179)	(0.055)	(0.024)
Descriptive×Harmful	___-0.011***___	-0.073*	-0.020	0.017	0.079	-0.044	-0.146	0.320	-0.091*	-0.032
	___(0.003)___	(0.035)	(0.040)	(0.045)	(0.101)	(0.073)	(0.097)	(0.187)	(0.044)	(0.023)
Injunctive×Harmful	-0.007*	___-0.160***___	0.025	-0.016	0.041	0.048	-0.074	0.163	-0.037	-0.016
	(0.003)	___(0.040)___	(0.032)	(0.035)	(0.092)	(0.086)	(0.078)	(0.164)	(0.041)	(0.023)
Empathy×Harmful	-0.002	-0.060	-0.019	0.042	0.002	0.067	0.049	0.247	-0.011	-0.014
	(0.003)	(0.045)	(0.037)	(0.060)	(0.091)	(0.072)	(0.073)	(0.171)	(0.044)	(0.026)
Deliberation×Harmful	-0.006*	-0.037	-0.009	0.055	0.100	0.175	-0.042	0.243	-0.106*	-0.011
	(0.003)	(0.042)	(0.034)	(0.044)	(0.132)	(0.097)	(0.095)	(0.153)	(0.053)	(0.024)
Constant	0.165***	-1.930***	-5.442***	-4.866***	-3.015***	-4.762***	-5.612***	-4.946***	-4.527***	-1.672***
	(0.018)	(0.140)	(0.286)	(0.321)	(0.261)	(0.448)	(0.421)	(0.399)	(0.278)	(0.138)
Observations	57134	57134	57134	57134	57134	57134	57134	57134	57134	57133

Standard errors in parentheses. * p<0.05, ** p<0.01, *** p<0.001.

Bonferroni correction for 14 tests (in bold), or 7 tests (in italic) for all the regressors.

Table S2: Model 1: linear regression with robust standard errors clustered at the participant and post level. From Models 2 to 9: logit regression with robust standard errors clustered at the participant and post level.

3 Overall Engagement: robustness check for topics

In Table S3, we check whether the posts' topics moderate the effects of the interaction between the conditions and the harmfulness of the topics for the overall engagement. We focus here only on the results found in the main analysis (see Table S2) - the interaction between the Cool and Descriptive conditions with the harmfulness of the posts and the topics. Assisted suicide and gun control topics moderate the effect of the interactions between the Cool and Descriptive conditions and the harmfulness of the posts. Specifically, the higher the harmfulness of the post the lower the overall engagement with the post. Moreover, legalisation and politics moderate the effect of the interaction between the Descriptive condition and the harmfulness of the posts, and in this case, the higher the harmfulness of the post the higher the overall engagement with the post.

	Abortion	Ass. suicide	Gun control	Legalization	Politics	Science	Social justice
Harmful	-0.005*	-0.005*	-0.005*	-0.003	-0.004	-0.006**	-0.007*
	(0.002)	(0.002)	(0.002)	(0.002)	(0.002)	(0.002)	(0.003)
Personal	0.064**	0.058**	0.066**	0.057**	0.061**	0.058**	0.066**
	(0.020)	(0.020)	(0.020)	(0.019)	(0.020)	(0.021)	(0.020)
Cool	0.053**	0.049*	0.054**	0.040*	0.047*	0.048*	0.048*
	(0.020)	(0.020)	(0.020)	(0.019)	(0.020)	(0.021)	(0.022)
Reputation	0.026	0.018	0.023	0.018	0.018	0.008	0.019
	(0.020)	(0.019)	(0.019)	(0.019)	(0.019)	(0.020)	(0.021)
Descriptive	0.125***	0.109***	0.119***	0.109***	0.116***	0.112***	0.118***
	(0.022)	(0.021)	(0.022)	(0.021)	(0.021)	(0.022)	(0.026)
Injunctive	0.084***	0.075***	0.084***	0.083***	0.078***	0.068**	0.085***
	(0.021)	(0.020)	(0.020)	(0.020)	(0.020)	(0.021)	(0.021)
Empathy	0.048*	0.040	0.048*	0.039*	0.042*	0.039	0.046*
	(0.020)	(0.020)	(0.020)	(0.019)	(0.020)	(0.021)	(0.022)
Deliberation	0.045*	0.038	0.047*	0.040*	0.040*	0.040	0.043*
	(0.020)	(0.020)	(0.020)	(0.019)	(0.020)	(0.021)	(0.020)
Personal×Harmful	-0.006*	-0.005	-0.006*	-0.005	-0.005	-0.004	-0.010*
	(0.003)	(0.003)	(0.003)	(0.003)	(0.003)	(0.003)	(0.004)
Cool×Harmful	-0.009**	-0.009**	-0.010***	-0.008**	-0.010***	-0.009**	-0.010*
	(0.003)	(0.003)	(0.003)	(0.003)	(0.003)	(0.003)	(0.004)
Reputation×Harmful	-0.005	-0.003	-0.004	-0.003	-0.003	-0.002	-0.006
	(0.003)	(0.003)	(0.003)	(0.003)	(0.003)	(0.003)	(0.004)
Descriptive×Harmful	-0.013***	-0.011***	-0.012***	-0.011***	-0.013***	-0.011***	-0.015**
	(0.003)	(0.003)	(0.003)	(0.003)	(0.003)	(0.003)	(0.005)
Injunctive×Harmful	-0.008**	-0.006*	-0.008*	-0.008**	-0.006*	-0.005	-0.012**
	(0.003)	(0.003)	(0.003)	(0.003)	(0.003)	(0.003)	(0.004)
Empathy×Harmful	-0.003	-0.001	-0.002	-0.001	-0.002	-0.000	-0.006
	(0.003)	(0.003)	(0.003)	(0.003)	(0.003)	(0.003)	(0.004)
Deliberation×Harmful	-0.006*	-0.005	-0.007*	-0.006	-0.006	-0.006	-0.008*
	(0.003)	(0.003)	(0.003)	(0.003)	(0.003)	(0.003)	(0.004)
Topic	-0.025*	-0.022	0.022	0.070***	-0.044***	-0.026*	0.037*
	(0.011)	(0.021)	(0.019)	(0.018)	(0.010)	(0.011)	(0.016)
Personal×Topic	-0.044	0.371**	0.046	-0.023	-0.151*	0.021	-0.002
	(0.027)	(0.123)	(0.047)	(0.047)	(0.061)	(0.026)	(0.025)
Cool×Topic	-0.054*	0.296**	0.036	-0.039	-0.015	-0.008	-0.000

	(0.024)	(0.113)	(0.043)	(0.048)	(0.053)	(0.023)	(0.023)
Reputation×Topic	-0.080***	0.149	0.094	-0.044	-0.080	0.045	0.006
	(0.024)	(0.111)	(0.050)	(0.046)	(0.058)	(0.026)	(0.025)
Descriptive×Topic	-0.100***	0.549***	0.134*	-0.142**	-0.194**	0.007	0.002
	(0.026)	(0.138)	(0.054)	(0.049)	(0.061)	(0.027)	(0.037)
Injunctive×Topic	-0.057*	0.432***	0.088*	-0.040	-0.109*	0.052*	-0.010
	(0.025)	(0.121)	(0.044)	(0.047)	(0.052)	(0.025)	(0.025)
Empathy×Topic	-0.065*	0.405**	0.042	-0.021	-0.125	0.040	0.004
	(0.026)	(0.126)	(0.047)	(0.047)	(0.067)	(0.025)	(0.024)
Deliberation×Topic	-0.047	0.444**	0.086	-0.034	-0.002	-0.006	0.002
	(0.026)	(0.136)	(0.047)	(0.043)	(0.048)	(0.024)	(0.034)
Personal×Harmful×Topic	0.012*	-0.532**	-0.100**	0.040	0.024*	-0.010	0.006
	(0.006)	(0.169)	(0.033)	(0.045)	(0.010)	(0.006)	(0.005)
Cool×Harmful×Topic	0.008	**-0.477****	**-0.092****	0.090	0.007	0.002	0.002
	(0.004)	**(0.154)**	**(0.030)**	(0.047)	(0.009)	(0.006)	(0.006)
Reputation×Harmful×Topic	0.014**	-0.241	-0.151***	0.048	0.014	-0.009	0.003
	(0.005)	(0.153)	(0.035)	(0.043)	(0.010)	(0.006)	(0.005)
Descriptive×Harmful×Topic	0.012*	**-0.762*****	**-0.178*****	**0.215*****	**0.036****	-0.001	0.004
	(0.005)	**(0.189)**	**(0.041)**	**(0.055)**	**(0.011)**	(0.007)	(0.006)
Injunctive×Harmful×Topic	0.012*	-0.610***	-0.142***	0.015	0.016	-0.011	0.008
	(0.006)	(0.166)	(0.032)	(0.045)	(0.009)	(0.006)	(0.005)
Empathy×Harmful×Topic	0.014**	-0.588***	-0.098**	0.042	0.023*	-0.018**	0.006
	(0.005)	(0.174)	(0.032)	(0.046)	(0.012)	(0.006)	(0.005)
Deliberation×Harmful×Topic	0.007	-0.635***	-0.146***	0.038	0.001	0.004	0.003
	(0.005)	(0.182)	(0.032)	(0.041)	(0.008)	(0.006)	(0.006)
Constant	0.168***	0.167***	0.162***	0.151***	0.165***	0.173***	0.161***
	(0.013)	(0.013)	(0.013)	(0.013)	(0.013)	(0.014)	(0.017)
Observations	57134	57134	57134	57134	57134	57134	57134

Standard errors in parentheses. * p<0.05, ** p<0.01, *** p<0.001.

Bonferroni correction for 7 tests in bold only for the interactions of interest.

Table S3: From Models 1 to 7: linear regressions with robust standard errors clustered at the participant and post levels.

4 Like: robustness check for topics

In Table S4, we check whether the posts' topics moderate the effects of the interaction between the conditions and the harmfulness of the topics for the like intention. We focus here only on the results found in the main analysis (see Table S2) - the interaction between the Injunctive condition with the harmfulness of the post and the topic. Assisted suicide, gun control and legalization moderate the effect of the interaction between the Injunctive condition and the harmfulness of the post. Specifically. for assisted suicide and gun control, the higher the harmfulness of the post the lower the like intention to the post, while, for legalization the higher the harmfulness of the post the higher the like intention to the post.

	Abortion	Ass. suicide	Gun control	Legalization	Politics	Science	Social justice
Harmful	-0.265***	-0.274***	-0.275***	-0.254***	-0.283***	-0.273***	-0.282***
	(0.036)	(0.029)	(0.037)	(0.040)	(0.045)	(0.038)	(0.037)
Personal	0.458**	0.440**	0.506***	0.424**	0.467**	0.390*	0.399*
	(0.149)	(0.136)	(0.142)	(0.154)	(0.145)	(0.155)	(0.167)
Cool	0.377**	0.383**	0.393**	0.332**	0.411**	0.365*	0.294
	(0.131)	(0.136)	(0.133)	(0.123)	(0.132)	(0.142)	(0.151)
Reputation	0.294*	0.297*	0.309*	0.269	0.275*	0.228	0.233
	(0.135)	(0.141)	(0.136)	(0.144)	(0.135)	(0.128)	(0.157)
Descriptive	0.625***	0.580***	0.657***	0.551***	0.599***	0.592***	0.552***
	(0.137)	(0.132)	(0.130)	(0.132)	(0.136)	(0.151)	(0.159)
Injunctive	0.640***	0.624***	0.665***	0.703***	0.671***	0.559***	0.612***
	(0.140)	(0.134)	(0.143)	(0.149)	(0.142)	(0.136)	(0.156)
Empathy	0.333*	0.307*	0.372*	0.298	0.323*	0.229	0.304
	(0.160)	(0.139)	(0.154)	(0.180)	(0.160)	(0.158)	(0.186)
Deliberation	0.326*	0.319*	0.381**	0.258	0.357*	0.344*	0.327*
	(0.139)	(0.138)	(0.134)	(0.143)	(0.140)	(0.151)	(0.152)
Personal×Harmful	-0.144**	-0.121**	-0.128**	-0.119*	-0.141**	-0.111*	-0.080
	(0.051)	(0.044)	(0.048)	(0.048)	(0.050)	(0.054)	(0.066)
Cool×Harmful	-0.066	-0.067	-0.065	-0.061	-0.104*	-0.080	-0.001
	(0.038)	(0.038)	(0.037)	(0.035)	(0.041)	(0.044)	(0.052)
Reputation×Harmful	-0.076*	-0.069	-0.066	-0.066	-0.059	-0.067	-0.045
	(0.038)	(0.044)	(0.038)	(0.039)	(0.042)	(0.040)	(0.049)
Descriptive×Harmful	-0.076*	-0.070	-0.081*	-0.068*	-0.071	-0.095*	-0.049
	(0.035)	(0.039)	(0.035)	(0.034)	(0.039)	(0.042)	(0.046)
Injunctive×Harmful	-0.154***	-0.155***	-0.157***	-0.174***	-0.188***	-0.162***	-0.128*
	(0.040)	(0.039)	(0.040)	(0.041)	(0.048)	(0.048)	(0.054)
Empathy×Harmful	-0.070	-0.058	-0.068	-0.060	-0.071	-0.047	-0.041
	(0.045)	(0.042)	(0.044)	(0.047)	(0.049)	(0.046)	(0.061)
Deliberation×Harmful	-0.039	-0.033	-0.042	-0.025	-0.059	-0.047	-0.008
	(0.042)	(0.040)	(0.042)	(0.042)	(0.047)	(0.047)	(0.056)
Topic	-0.644	-0.051	-0.111	0.378*	0.211	-0.154	0.244
	(0.348)	(0.199)	(0.411)	(0.175)	(0.323)	(0.221)	(0.249)
Personal×Topic	-0.367	1.772*	0.205	-0.226	-2.064	0.245	0.083
	(0.281)	(0.788)	(0.381)	(0.439)	(1.537)	(0.254)	(0.301)
Cool×Topic	-0.390	2.727**	0.377	-0.425	-1.592	-0.049	0.149

	(0.320)	(0.930)	(0.377)	(0.427)	(1.776)	(0.234)	(0.248)
Reputation×Topic	-0.550*	1.898*	0.513	-0.463*	-2.078	0.185	0.105
	(0.267)	(0.962)	(0.383)	(0.214)	(1.102)	(0.339)	(0.221)
Descriptive×Topic	-0.855*	2.347**	0.139	-0.559	-1.488*	-0.040	0.111
	(0.388)	(0.806)	(0.367)	(0.419)	(0.690)	(0.230)	(0.245)
Injunctive×Topic	-0.307	2.251**	0.475	-0.641***	-1.931	0.352	0.021
	(0.447)	(0.735)	(0.370)	(0.181)	(2.004)	(0.233)	(0.264)
Empathy×Topic	-0.640	3.906***	0.089	-0.473	-0.681	0.383	-0.036
	(0.471)	(0.996)	(0.410)	(0.371)	(1.548)	(0.332)	(0.290)
Deliberation×Topic	-0.062	3.208***	0.278	-0.165	-1.128	-0.111	-0.075
	(0.418)	(0.911)	(0.384)	(0.250)	(1.446)	(0.233)	(0.247)
Personal×Harmful×Topic	0.246**	-2.710*	-1.123**	0.375	0.415	-0.054	-0.097
	(0.089)	(1.181)	(0.396)	(0.327)	(0.300)	(0.104)	(0.089)
Cool×Harmful×Topic	0.096	-4.546**	-0.839***	0.683*	0.380	0.090	-0.152
	(0.077)	(1.497)	(0.182)	(0.335)	(0.339)	(0.087)	(0.087)
Reputation×Harmful×Topic	0.199**	-3.298*	-1.295***	0.606***	0.338	0.016	-0.041
	(0.065)	(1.489)	(0.206)	(0.163)	(0.215)	(0.113)	(0.069)
Descriptive×Harmful×Topic	0.175	-3.475**	-0.928***	0.939**	0.251	0.112	-0.053
	(0.112)	(1.229)	(0.247)	(0.331)	(0.146)	(0.071)	(0.072)
Injunctive×Harmful×Topic	0.055	-3.342**	-1.080**	0.530**	0.417	0.000	-0.068
	(0.146)	(1.124)	(0.346)	(0.173)	(0.383)	(0.096)	(0.101)
Empathy×Harmful×Topic	0.206*	-6.234***	-0.890***	0.679*	0.153	-0.070	-0.030
	(0.089)	(1.623)	(0.114)	(0.268)	(0.312)	(0.108)	(0.081)
Deliberation×Harmful×Topic	0.042	-4.870***	-1.039***	0.497**	0.259	0.070	-0.051
	(0.103)	(1.432)	(0.077)	(0.158)	(0.278)	(0.069)	(0.076)
Constant	-1.874***	-1.923***	-1.913***	-2.028***	-1.921***	-1.893***	-1.976***
	(0.134)	(0.100)	(0.140)	(0.170)	(0.139)	(0.154)	(0.146)
Observations	57134	57134	57134	57134	57134	57134	57134

Standard errors in parentheses. * p<0.05, ** p<0.01, *** p<0.001.

Bonferroni correction for 7 tests in bold only for the interactions of interest.

Table S4: Model 1: logit regression with robust standard errors clustered at the participant and post level. Model 2: logit regression with robust standard errors clustered at the participant level. From Models 3 to 7 logit regression with robust standard errors clustered at the participant and post level.

5 Demographics: robustness check

In Table S5, we check whether gender and age moderate the effects of the interaction between the conditions and the harmfulness of the topics for both the overall engagement and the intention to like. We focus here only on the results found in the main analysis (see Table S2). For the overall engagement, we focus on the interaction between the Cool and Descriptive conditions with the harmfulness of the post and the demographic information. For the intention to like, we focus on the interaction between the Injunctive condition with the harmfulness of the post and the demographic information. For both the overall engagement and the intention to like, gender and age do not moderate the effect of the interactions between the conditions and the harmfulness of the post.

	Engagement Female	Like Female	Engagement Age	Like Age
Harmful	-0.003	-0.219***	-0.003	-0.288**
	(0.003)	(0.042)	(0.008)	(0.093)
Personal	0.040	0.254	0.078	0.678
	(0.027)	(0.194)	(0.060)	(0.389)
Cool	0.031	0.380*	0.078	0.666
	(0.031)	(0.191)	(0.058)	(0.419)
Reputation	-0.006	0.205	0.036	0.365
	(0.029)	(0.189)	(0.061)	(0.463)
Descriptive	0.142***	0.815***	0.185**	0.731
	(0.034)	(0.181)	(0.062)	(0.382)
Injunctive	0.011	0.382*	0.088	0.564
	(0.030)	(0.193)	(0.061)	(0.414)
Empathy	-0.010	0.085	0.030	0.463
	(0.027)	(0.196)	(0.062)	(0.406)
Deliberation	0.023	0.282	0.007	0.380
	(0.033)	(0.219)	(0.063)	(0.445)
Personal×Harmful	-0.004	-0.096	-0.004	-0.195
	(0.004)	(0.059)	(0.010)	(0.142)
Cool×Harmful	-0.010*	-0.122*	-0.017*	-0.155
	(0.004)	(0.051)	(0.008)	(0.112)
Reputation×Harmful	-0.001	-0.053	0.001	0.064
	(0.004)	(0.051)	(0.010)	(0.158)
Descriptive×Harmful	-0.018***	-0.156**	-0.010	0.019
	(0.005)	(0.048)	(0.010)	(0.100)
Injunctive×Harmful	-0.003	-0.164**	-0.004	-0.138
	(0.004)	(0.054)	(0.009)	(0.116)
Empathy×Harmful	-0.000	-0.037	-0.008	-0.050
	(0.004)	(0.050)	(0.010)	(0.116)
Deliberation×Harmful	-0.009	-0.068	-0.008	-0.121
	(0.005)	(0.063)	(0.009)	(0.128)
Demographic	-0.019	-0.064	-0.000	0.001
	(0.030)	(0.240)	(0.001)	(0.009)
Harmful×Demographic	-0.003	-0.113	-0.000	0.000
	(0.005)	(0.060)	(0.000)	(0.002)

Personal×Demographic	0.047	0.483	-0.001	-0.006
	(0.039)	(0.263)	(0.002)	(0.010)
Cool×Demographic	0.039	0.059	-0.001	-0.008
	(0.045)	(0.293)	(0.002)	(0.011)
Reputation×Demographic	0.050	0.235	-0.001	-0.002
	(0.040)	(0.294)	(0.002)	(0.012)
Descriptive×Demographic	-0.052	-0.393	-0.002	-0.003
	(0.042)	(0.239)	(0.002)	(0.009)
Injunctive×Demographic	0.132**	0.512*	-0.000	0.002
	(0.040)	(0.255)	(0.002)	(0.010)
Empathy×Demographic	0.098*	0.491	0.000	-0.004
	(0.043)	(0.290)	(0.002)	(0.010)
Deliberation×Demographic	0.034	0.169	0.001	-0.001
	(0.042)	(0.288)	(0.002)	(0.011)
Personal×Harmful×Demographic	-0.004	-0.090	-0.000	0.002
	(0.005)	(0.082)	(0.000)	(0.003)
Cool×Harmful×Demographic	-0.001	0.101	0.000	0.002
	(0.006)	(0.075)	(0.000)	(0.003)
Reputation×Harmful×Demographic	-0.004	-0.030	-0.000	-0.004
	(0.006)	(0.083)	(0.000)	(0.004)
Descriptive×Harmful×Demographic	0.012*	0.176**	-0.000	-0.003
	(0.005)	(0.068)	(0.000)	(0.002)
Injunctive×Harmful×Demographic	-0.008	0.026	-0.000	-0.001
	(0.006)	(0.066)	(0.000)	(0.003)
Empathy×Harmful×Demographic	-0.003	-0.069	0.000	-0.000
	(0.006)	(0.076)	(0.000)	(0.003)
Deliberation×Harmful×Demographic	0.006	0.064	0.000	0.002
	(0.006)	(0.075)	(0.000)	(0.003)
Constant	0.178***	-1.889***	0.178***	-1.943***
	(0.022)	(0.169)	(0.043)	(0.341)
Observations	53197	53197	54877	54877

Standard errors in parentheses. * p<0.05, ** p<0.01, *** p<0.001.

Bonferroni correction for 7 tests in bold only for the interactions of interest.

Table S5: Models 1 and 3: linear regressions with robust standard errors clustered at the participant and post level. Models 2 and 4: logit regressions with robust standard errors clustered at the participant and post level.

6 Political orientation: robustness check

In Table S6, we check whether political orientation moderates the effects of the interaction between the conditions and the harmfulness of the topics for both the overall engagement and the like intention. We focus here only on the results found in the main analysis (see Table S2). For the overall engagement, we focus on the interaction between the Cool and Descriptive conditions with the harmfulness of the post and the demographic information. For the like intention, we focus on the interaction between the Injunctive condition with the harmfulness of the post and the demographic information. For both the overall engagement and the like intention, the political orientation does not moderate the effect of the interactions between the conditions and the harmfulness of the post.

	Engagement Dems	Like Dems	Engagement Reps	Like Reps	Engagement Indeps	Like Indeps
Harmful	-0.002	-0.239***	-0.006	-0.309***	-0.004	-0.262***
	(0.003)	(0.048)	(0.003)	(0.047)	(0.003)	(0.040)
Personal	0.056*	0.266	0.052*	0.405**	0.075**	0.590***
	(0.025)	(0.218)	(0.022)	(0.145)	(0.023)	(0.162)
Cool	0.073**	0.506**	0.030	0.244	0.060*	0.489**
	(0.025)	(0.163)	(0.021)	(0.129)	(0.023)	(0.152)
Reputation	0.057*	0.479*	0.000	0.220	0.017	0.248
	(0.025)	(0.207)	(0.020)	(0.139)	(0.022)	(0.149)
Descriptive	0.143***	0.703***	0.105***	0.507***	0.091***	0.612***
	(0.030)	(0.189)	(0.025)	(0.137)	(0.025)	(0.162)
Injunctive	0.060*	0.593**	0.082***	0.603***	0.091***	0.728***
	(0.025)	(0.200)	(0.022)	(0.135)	(0.023)	(0.160)
Empathy	0.046*	0.247	0.034	0.289	0.052*	0.383*
	(0.023)	(0.201)	(0.022)	(0.156)	(0.024)	(0.172)
Deliberation	0.059*	0.398*	0.031	0.291*	0.040	0.330*
	(0.025)	(0.189)	(0.022)	(0.146)	(0.023)	(0.157)
Personal×Harmful	-0.007	-0.087	-0.006	-0.116*	-0.006	-0.144*
	(0.004)	(0.063)	(0.003)	(0.057)	(0.003)	(0.058)
Cool×Harmful	-0.009*	-0.071	-0.007*	-0.032	-0.011**	-0.080*
	(0.004)	(0.049)	(0.003)	(0.039)	(0.003)	(0.040)
Reputation×Harmful	-0.002	-0.056	-0.002	-0.077	-0.006	-0.073
	(0.004)	(0.061)	(0.003)	(0.043)	(0.003)	(0.042)
Descriptive×Harmful	-0.008*	-0.057	-0.012**	-0.050	-0.013***	-0.106**
	(0.004)	(0.051)	(0.004)	(0.040)	(0.003)	(0.040)
Injunctive×Harmful	-0.010*	-0.163**	-0.008*	-0.138***	-0.007*	-0.157***
	(0.004)	(0.061)	(0.003)	(0.041)	(0.003)	(0.043)
Empathy×Harmful	-0.001	0.013	-0.002	-0.084	-0.002	-0.076
	(0.003)	(0.057)	(0.003)	(0.049)	(0.004)	(0.052)
Deliberation×Harmful	-0.004	-0.004	-0.006	-0.045	-0.007*	-0.030
	(0.003)	(0.057)	(0.003)	(0.050)	(0.003)	(0.049)
Political Orientation	0.094**	0.557**	-0.113***	-1.371***	-0.000	0.124
	(0.030)	(0.198)	(0.031)	(0.305)	(0.029)	(0.218)
Harmful×PO	-0.005	-0.059	0.007	0.314***	-0.005	-0.045
	(0.004)	(0.069)	(0.005)	(0.071)	(0.004)	(0.078)

Personal×PO	0.016	0.360	0.066	0.745*	-0.063	-0.651*
	(0.044)	(0.294)	(0.048)	(0.329)	(0.042)	(0.300)
Cool×PO	-0.051	-0.218	0.134**	1.328***	-0.048	-0.484
	(0.039)	(0.247)	(0.047)	(0.372)	(0.040)	(0.259)
Reputation×PO	-0.081*	-0.350	0.129*	0.995*	0.001	0.108
	(0.037)	(0.279)	(0.049)	(0.393)	(0.043)	(0.304)
Descriptive×PO	-0.058	-0.152	0.077	1.094**	0.082	-0.063
	(0.043)	(0.236)	(0.051)	(0.348)	(0.049)	(0.303)
Injunctive×PO	0.043	0.106	-0.011	0.648	-0.052	-0.356
	(0.040)	(0.267)	(0.041)	(0.337)	(0.043)	(0.296)
Empathy×PO	-0.011	0.144	0.047	0.578	-0.036	-0.273
	(0.038)	(0.232)	(0.049)	(0.415)	(0.040)	(0.258)
Deliberation×PO	-0.033	-0.042	0.077	0.854*	-0.001	-0.003
	(0.038)	(0.244)	(0.048)	(0.356)	(0.043)	(0.275)
Personal×Harmful×PO	0.003	-0.070	0.000	-0.126	0.002	0.099
	(0.006)	(0.098)	(0.007)	(0.081)	(0.006)	(0.126)
Cool×Harmful×PO	0.001	0.003	-0.011	-0.287***	0.008	0.066
	(0.005)	(0.065)	(0.006)	(0.077)	(0.005)	(0.080)
Reputation×Harmful×PO	-0.004	-0.036	-0.007	-0.097	0.013	0.036
	(0.005)	(0.082)	(0.006)	(0.093)	(0.007)	(0.104)
Descriptive×Harmful×PO	-0.007	-0.048	-0.001	-0.227**	0.009	0.116
	(0.005)	(0.065)	(0.007)	(0.086)	(0.006)	(0.093)
Injunctive×Harmful×PO	0.005	0.003	0.002	-0.197*	-0.000	-0.033
	(0.006)	(0.083)	(0.006)	(0.098)	(0.006)	(0.105)
Empathy×Harmful×PO	-0.002	-0.163	0.007	0.030	0.002	0.066
	(0.006)	(0.086)	(0.007)	(0.083)	(0.006)	(0.101)
Deliberation×Harmful×PO	-0.004	-0.094	-0.002	-0.087	0.006	-0.024
	(0.005)	(0.085)	(0.006)	(0.080)	(0.006)	(0.088)
Constant	0.119***	-2.230***	0.179***	-1.800***	0.165***	-1.960***
	(0.016)	(0.154)	(0.020)	(0.144)	(0.021)	(0.162)
Observations	57134	57134	57134	57134	57134	57134

Standard errors in parentheses. * p<0.05, ** p<0.01, *** p<0.001.

Bonferroni correction for 7 tests in bold only for the interactions of interest.

Table S6: Models 1, 3 and 5: linear regressions with robust standard errors clustered at the participant and post level. Models 2, 4 and 6: logit regressions with robust standard errors clustered at the participant and post level.

7 Text Analysis

7.1 Definition of the metrics

The following definitions are taken from the PeRspective API website.

Toxicity. A rude, disrespectful, or unreasonable comment that is likely to make people leave a discussion.

Severe toxicity. A very hateful, aggressive, disrespectful comment or otherwise very likely to make a user leave a discussion or give up on sharing their perspective. This attribute is much less sensitive to more mild forms of toxicity, such as comments that include positive uses of curse words.

Identity attack. Insulting, inflammatory, or negative comment towards a person or a group of people.

Profanity. Swear words, curse words, or other obscene or profane language.

Threat. Describes an intention to inflict pain, injury, or violence against an individual or group.

Sexually explicit. Contains references to sexual acts, body parts, or other lewd content.

Flirtation. Pickup lines, complimenting appearance, subtle sexual innuendos, etc.

Attack on the author. Attack on the author of an article or post.

Attack on commenter. Attack on fellow commenter.

Incoherent. Difficult to understand, nonsensical.

Inflammatory. Intending to provoke or inflame.

Likely to reject. Overall measure of the likelihood for the comment to be rejected according to the NYT's moderation.

Obscene. Obscene or vulgar language such as cursing.

Spam. Irrelevant and unsolicited commercial content.

Unsubstantial. Trivial or short comments.

7.2 Analysis

We conducted a series of exploratory analyses to test for differences in the style of the comments left in each condition (Tables S7 to S9). Models that detect the harmful intent of comments (e.g., identity attacks, insults, threats) show no significant differences or weakly significant differences ($p > 0.01$) that do not survive correction for multiple comparisons. However, two metrics show a more consistent pattern: comment substance and coherence. Several interventions show a significant interaction between post harmfulness and experimental condition: compared to the control group, substance and coherence tend to increase as the original content becomes more harmful. For both metrics, one intervention survives multiple comparisons: highlighting reputation. In Table S7 we report the regression analysis regarding these two metrics. The regression tables of the other metrics are reported in Tables S8 and S9.

	Unsubstantial	Incoherent
Harmful	0.108*	0.058
	(0.049)	(0.062)
Personal	0.097	0.079
	(0.053)	(0.051)
Cool	0.140*	0.043
	(0.056)	(0.040)
Reputation	0.126*	0.152*

	(0.060)	(0.059)
Descriptive	0.147**	0.108
	(0.049)	(0.056)
Injunctive	0.088	0.056
	(0.052)	(0.046)
Empathy	0.098	0.112*
	(0.054)	(0.048)
Deliberation	0.124*	0.089*
	(0.053)	(0.043)
Personal×Harmful	-0.184*	-0.134
	(0.074)	(0.093)
Cool×Harmful	-0.178*	-0.028
	(0.068)	(0.069)
Reputation×Harmful	-0.249***	-0.298***
	(0.065)	(0.080)
Descriptive×Harmful	-0.205*	-0.271**
	(0.077)	(0.096)
Injunctive×Harmful	-0.131	-0.137
	(0.078)	(0.085)
Empathy×Harmful	-0.156*	-0.230*
	(0.076)	(0.088)
Deliberation×Harmful	-0.191**	-0.202**
	(0.071)	(0.073)
Constant	0.635***	0.379***
	(0.040)	(0.039)
Observations	1560	1560

Standard errors in parentheses

* $p < 0.05$, ** $p < 0.01$, *** $p < 0.001$

Table S7: Linear regressions with robust standard errors clustered at the participant and post level.

	Severe Toxicity	Identity Attack	Insult	Profanity	Sexually Explicit	Threat	Flirtation
Harmful	0.063**	0.081***	0.246***	0.192**	0.051***	0.014	0.065*
	(0.021)	(0.017)	(0.054)	(0.057)	(0.014)	(0.024)	(0.029)
Personal	-0.006	0.030*	-0.012	-0.003	0.013	0.024	-0.005
	(0.012)	(0.012)	(0.033)	(0.030)	(0.011)	(0.014)	(0.024)
Cool	-0.015	-0.006	-0.009	-0.016	-0.012	0.014	0.005
	(0.012)	(0.021)	(0.048)	(0.032)	(0.013)	(0.021)	(0.027)
Reputation	-0.026	-0.005	-0.017	-0.016	-0.012	-0.015	0.038
	(0.015)	(0.016)	(0.042)	(0.031)	(0.009)	(0.011)	(0.031)
Descriptive	-0.016	-0.006	-0.036	-0.024	-0.012	-0.006	0.007
	(0.012)	(0.015)	(0.024)	(0.029)	(0.008)	(0.011)	(0.022)
Injunctive	-0.004	-0.000	0.017	0.024	0.004	0.001	0.027
	(0.011)	(0.018)	(0.032)	(0.031)	(0.009)	(0.010)	(0.028)
Empathy	-0.020	-0.002	-0.039	-0.026	-0.006	-0.002	0.004
	(0.013)	(0.017)	(0.035)	(0.033)	(0.012)	(0.011)	(0.018)
Deliberation	0.002	0.005	0.025	0.028	0.007	0.028*	0.008
	(0.013)	(0.015)	(0.036)	(0.033)	(0.011)	(0.012)	(0.024)
Personal×Harmful	0.002	-0.027	0.015	-0.018	-0.018	-0.041	-0.002
	(0.029)	(0.020)	(0.073)	(0.062)	(0.025)	(0.028)	(0.042)
Cool×Harmful	0.025	0.039	-0.007	0.004	0.040	-0.004	-0.018
	(0.025)	(0.037)	(0.100)	(0.069)	(0.030)	(0.039)	(0.046)
Reputation×Harmful	0.066	0.018	0.065	0.079	0.050*	0.018	-0.077
	(0.038)	(0.028)	(0.086)	(0.067)	(0.022)	(0.031)	(0.056)
Descriptive×Harmful	0.028	-0.003	0.077	0.063	0.025	0.007	-0.033
	(0.028)	(0.033)	(0.065)	(0.075)	(0.022)	(0.026)	(0.036)
Injunctive×Harmful	0.023	0.024	0.002	-0.039	0.004	0.016	-0.067
	(0.030)	(0.038)	(0.075)	(0.076)	(0.022)	(0.027)	(0.041)
Empathy×Harmful	0.032	0.035	0.055	0.030	0.016	-0.001	0.001
	(0.028)	(0.027)	(0.084)	(0.069)	(0.024)	(0.024)	(0.033)
Deliberation×Harmful	-0.005	0.011	-0.012	-0.067	-0.009	-0.041	-0.050
	(0.028)	(0.019)	(0.071)	(0.063)	(0.018)	(0.028)	(0.038)
Constant	0.003	0.012	0.042	0.030	0.016*	0.022*	0.245***
	(0.011)	(0.011)	(0.026)	(0.026)	(0.007)	(0.010)	(0.018)
Observations	1560	1560	1560	1560	1560	1560	1560

Standard errors in parentheses

* $p < 0.05$, ** $p < 0.01$, *** $p < 0.001$

Table S8: Linear regressions with robust standard errors clustered at the participant and post level.

	Attack on Aut	Attack on Comm	Toxicity	Inflammatory	Likely to Reject	Obscene	Spam
Harmful	0.256***	0.425***	0.331***	0.166**	0.401***	0.295***	0.001
	(0.019)	(0.095)	(0.073)	(0.050)	(0.074)	(0.075)	(0.024)
Personal	0.023	-0.021	0.009	0.047	0.040	0.040	-0.008
	(0.034)	(0.061)	(0.041)	(0.050)	(0.062)	(0.034)	(0.026)
Cool	0.031	0.031	-0.005	0.001	0.120*	0.013	0.003
	(0.027)	(0.068)	(0.051)	(0.054)	(0.058)	(0.038)	(0.022)
Reputation	0.045	0.037	-0.017	0.059	0.110	0.075	0.026
	(0.036)	(0.070)	(0.047)	(0.053)	(0.070)	(0.041)	(0.015)
Descriptive	0.025	-0.095	-0.048	-0.032	0.029	0.013	0.051
	(0.030)	(0.055)	(0.034)	(0.038)	(0.059)	(0.034)	(0.047)
Injunctive	0.008	-0.030	0.019	0.034	0.079	0.067	0.008
	(0.024)	(0.061)	(0.038)	(0.049)	(0.061)	(0.039)	(0.022)
Empathy	-0.005	-0.034	-0.051	0.014	0.003	0.045	0.022
	(0.023)	(0.054)	(0.043)	(0.046)	(0.068)	(0.041)	(0.026)
Deliberation	0.001	-0.071	0.035	0.060	0.082	0.087	-0.011
	(0.031)	(0.056)	(0.043)	(0.039)	(0.060)	(0.047)	(0.024)
Personal×Harm	-0.099	-0.046	-0.056	-0.068	-0.098	-0.137	0.038
	(0.062)	(0.101)	(0.084)	(0.070)	(0.088)	(0.080)	(0.045)
Cool×Harm	-0.105*	-0.098	-0.025	-0.012	-0.152	-0.046	0.002
	(0.045)	(0.118)	(0.100)	(0.080)	(0.079)	(0.102)	(0.035)
Reputation×Harm	-0.097	-0.022	0.045	-0.085	-0.068	-0.048	-0.042
	(0.072)	(0.102)	(0.095)	(0.085)	(0.095)	(0.080)	(0.023)
Descriptive×Harm	-0.048	0.050	0.069	-0.038	0.055	-0.036	-0.094
	(0.041)	(0.103)	(0.083)	(0.061)	(0.081)	(0.092)	(0.072)
Injunctive×Harm	-0.005	0.063	-0.026	-0.034	-0.058	-0.170	-0.034
	(0.039)	(0.112)	(0.090)	(0.078)	(0.083)	(0.109)	(0.028)
Empathy×Harm	-0.005	0.006	0.057	-0.053	0.000	-0.117	-0.055
	(0.041)	(0.092)	(0.098)	(0.074)	(0.090)	(0.087)	(0.036)
Deliberation×Harm	-0.075	0.067	-0.064	-0.109*	-0.123	-0.223*	-0.001
	(0.072)	(0.087)	(0.084)	(0.042)	(0.084)	(0.085)	(0.033)
Constant	0.068***	0.270***	0.077*	0.234***	0.474***	0.020	0.070***
	(0.017)	(0.053)	(0.035)	(0.036)	(0.056)	(0.029)	(0.020)
Observations	1560	1560	1560	1560	1560	1560	1560

Standard errors in parentheses

* $p < 0.05$, ** $p < 0.01$, *** $p < 0.001$

Table S9: Linear regressions with robust standard errors clustered at the participant and post level. Attack on Aut = attack on author of the post; Attack on Comm = attack on another commenter of the post
